# Investigating Focal Connectivity Deficits in Alzheimer's Disease Using Directional Brain Networks Derived from Resting-State fMRI

**DOI:** 10.3389/fnagi.2017.00211

**Published:** 2017-07-06

**Authors:** Sinan Zhao, D Rangaprakash, Archana Venkataraman, Peipeng Liang, Gopikrishna Deshpande

**Affiliations:** ^1^AU MRI Research Center, Department of Electrical and Computer Engineering, Auburn UniversityAuburn, AL, United States; ^2^Department of Psychiatry and Biobehavioral Sciences, University of California, Los AngelesLos Angeles, CA, United States; ^3^Department of Electrical and Computer Engineering, Johns Hopkins UniversityBaltimore, MD, United States; ^4^Department of Radiology, Xuanwu Hospital, Capital Medical UniversityBeijing, China; ^5^Beijing Key Laboratory of Magnetic Resonance Imaging and Brain InformaticsBeijing, China; ^6^Key Laboratory for Neurodegenerative Diseases, Ministry of EducationBeijing, China; ^7^Department of Psychology, Auburn UniversityAuburn, AL, United States; ^8^Alabama Advanced Imaging Consortium, Auburn University and University of Alabama BirminghamAuburn, AL, United States

**Keywords:** Alzheimer's disease, functional MRI, effective connectivity, disease foci, brain stem, orbitofrontal cortex

## Abstract

Connectivity analysis of resting-state fMRI has been widely used to identify biomarkers of Alzheimer's disease (AD) based on brain network aberrations. However, it is not straightforward to interpret such connectivity results since our understanding of brain functioning relies on regional properties (activations and morphometric changes) more than connections. Further, from an interventional standpoint, it is easier to modulate the activity of regions (using brain stimulation, neurofeedback, etc.) rather than connections. Therefore, we employed a novel approach for identifying focal directed connectivity deficits in AD compared to healthy controls. In brief, we present a model of directed connectivity (using Granger causality) that characterizes the coupling among different regions in healthy controls and Alzheimer's disease. We then characterized group differences using a (between-subject) generative model of pathology, which generates latent connectivity variables that best explain the (within-subject) directed connectivity. Crucially, our generative model at the second (between-subject) level explains connectivity in terms of local or regionally specific abnormalities. This allows one to explain disconnections among multiple regions in terms of regionally specific pathology; thereby offering a target for therapeutic intervention. Two foci were identified, locus coeruleus in the brain stem and right orbitofrontal cortex. Corresponding disrupted connectivity network associated with the foci showed that the brainstem is the critical focus of disruption in AD. We further partitioned the aberrant connectomic network into four unique sub-networks, which likely leads to symptoms commonly observed in AD. Our findings suggest that fMRI studies of AD, which have been largely cortico-centric, could in future investigate the role of brain stem in AD.

## Introduction

Alzheimer's disease (AD) is a progressive neurodegenerative disorder with a long pre-morbid asymptomatic period (Caselli et al., 2004) which affects millions of elderly individuals worldwide (Blennow et al., 2006). The disease is initially characterized by the presence of neuronal and synaptic loss, β-amyloid (Aβ) production which results in the formation of intracellular neurofibrillary tangles and senile plaques (Buerger et al., 2006), thereby resulting in memory loss, cognitive decline, etc. Structural and functional decline are inevitable with age and the existing treatment options for AD are highly limited. Therefore, determining neural aberrations underlying AD are an important step in addressing this challenge.

Resting-state functional magnetic resonance imaging (RS-fMRI) is a promising neuroimaging technique that can non-invasively characterize underlying brain networks. This technology has been widely used to identify biomarkers of AD based on brain network alterations (Wang et al., 2007; Agosta et al., 2012; Sui et al., 2015). Seed-based approaches (Fox et al., 2009), independent components analysis (ICA) based approaches (Lee et al., 2015) and graph theory (Zhang et al., 2011) have been the three primary methods used in the study of resting-state functional connectivity (FC) in the brain. The seed-based approach involves predefining a region of interest (ROI) and extracting the BOLD signal from it; then a map of FC is obtained by calculating the cross-correlation between the time series extracted from the seed ROI and all other voxels in the brain. Previous studies in AD employing seed-based FC revealed decreased connectivity between the posterior cingulate cortex seed and regions spread across the whole brain in subjects with AD compared to healthy aging, with the Default Mode Network (DMN) being the most affected system (Zhang et al., 2009; Dennis and Thompson, 2014). Rather than define prior seeds, the ICA approach is model-free, which identifies independent components or co-activation networks throughout the brain. Damoiseaux et al. (2012) examined the components corresponding to the DMN for AD patients, and found significantly decreased FC in the posterior DMN and increased connectivity in ventral and anterior DMN in the AD group. Graph theoretic analysis is typically performed using FC matrices, revealing the topological properties and organization of the underlying brain network. For example, Brier et al. (2014) found that AD impacted the clustering coefficient and modularity in resting-state networks before the onset of the symptoms, suggesting that there might be a network-level pathology even in the preclinical stage. In summary, a profile of decreased connectivity has been consistently observed in AD.

However, most of the existing works on connectivity analyses have relied on FC or co-activation patterns, the literature on directed or effective connectivity (EC) patterns in AD is comparatively limited (more on this in the next paragraph). It is noteworthy that synchronization and causality in fMRI time series both represent distinct mechanisms in the brain (Friston, 2011), hence investigating EC aberrations in AD deserves attention. Motivated by this, we employed EC modeling to investigate aberrations in causal relationships between brain regions in AD. EC is often obtained using either of the two popular approaches, Granger causality (GC) (Granger, 1969; Deshpande et al., 2008, 2010a) and dynamic causal modeling (DCM) (Friston et al., 2003). DCM is highly dependent on prior assumptions concerning the underlying connectomic architecture and is therefore not generally considered suitable for analyses of large graphs. On the other hand, GC is a data-driven approach that does not need a predefined model (Deshpande et al., 2012; Sathian et al., 2013; Grant et al., 2014; Kapogiannis et al., 2014; Lacey et al., 2014; Wheelock et al., 2014; Chattaraman et al., 2016). Recent developments have demonstrated that GC is a viable technique for obtaining EC networks from fMRI data (Katwal et al., 2013; Wen et al., 2013). Therefore, in this study, we used a GC-based analysis framework. Strictly speaking, GC measures directed functional connectivity because it does not appeal to an underlying model of causal influences. In other words, GC tests for temporal precedence, thereby endowing functional connectivity with a direction. However, to emphasize the distinction between directed and non-directed connectivity, we will refer to our GC measures as effective connectivity (see Friston et al., 2013) for further discussion on this issue).

There have been several studies investigating EC-related aberrations in AD (Liu et al., 2012; Li et al., 2013; Chen et al., 2014; Zhong et al., 2014). These studies have reported distributed increases as well as decreases in directed relationships among brain regions in AD compared to healthy controls. However, these studies performing conventional GC analysis assume connectivity to be stationary over time, wherein only one connectivity value is obtained from the whole scan (Hampstead et al., 2011; Krueger et al., 2011; Lacey et al., 2011; Preusse et al., 2011; Sathian et al., 2011; Strenziok et al., 2011). However, connectivity, specifically the non-directed FC, has been shown to be non-stationary across time (Chang and Glover, 2010; Hutchison et al., 2013). Recent works suggests that connectivity varies over time, and that the temporal variability of connectivity is sensitive to human behavior in health and disease (Garrett et al., 2013; Jia et al., 2014; Rashid et al., 2016; Rangaprakash et al., 2017). Therefore, in addition to studying the conventional static effective connectivity (SEC), we also estimated dynamic effective connectivity (DEC; Grant et al., 2015; Hutcheson et al., 2015; Bellucci et al., 2016; Feng et al., 2016; Hampstead et al., 2016) from the resting-state fMRI data acquired from participants with AD as well as healthy controls (HC).

Traditionally, univariate statistical tests are performed for analyzing connectivity differences in population studies. Based on the statistical score, connectivity paths that differ from HC are ascertained. However, it is not straightforward to interpret such connectivity results, because traditionally our knowledge of brain functioning relies more on region-based properties (activations and morphometric changes) than connectivities. Further, from an interventional standpoint, it is easier to modulate the activity of brain regions (using brain stimulation, neurofeedback, etc.) rather than connections. With these viewpoints, Venkataraman et al. (2013) recently introduced a technique for identification of focal regions of functional disruption based on non-directed FC differences between populations. In this work, we extend this technique for identifying focal regions of disruption based on static as well as dynamic directed/effective connectivity aberrations in AD compared to HC.

We constructed brain networks using strength (SEC) and temporal variability (variance of DEC [vDEC]). After certain modifications to the connectivity measures, we fed them into the foci-identification model to obtain disrupted foci. The foci obtained independently from SEC and vDEC networks were then overlapped (intersection) to identify the common foci which exhibited impairments in both static and time-varying EC. Reduced temporal variance in dynamic connectivity is often associated with psychiatric disorders (Miller et al., 2016; Rangaprakash et al., 2017), and a relatively low variability of connectivity has been associated with poor behavioral performance in healthy individuals (Jia et al., 2014). Recall that a profile of decreased static connectivity has been consistently found in AD as discussed above. Taken together, we hypothesized that AD is characterized by dysfunctional disease foci, and that these foci are associated with connectivity paths that exhibit lower strength (SEC) as well as lower variability (vDEC) of effective connectivity.

## Materials and methods

### Participants

Data used in this study were obtained from the ADNI database (http://www.loni.ucla.edu/ADNI). Resting state fMRI data of 30 participants diagnosed with Alzheimer's disease (AD), along with 39 matched healthy controls (HC) were obtained through ADNI-2 cohort. Participants in this study were recruited between 2011 and 2013 through the ADNI-2 protocol, and we selected participants who had completed both 3D MPRAGE and resting-state fMRI data. Functional MRI data were obtained from a 3.0 Tesla Philips MR scanner with repetition time (TR) = 3,000 ms, echo time (TE) = 30 ms, flip angle (FA) = 80 degrees, field of view (FOV): RL (right-left) = 212, AP (anterior-posterior) = 198.75 mm, FH (foot-head) = 159 mm, voxel size: RL = 3.3125 mm, AP = 3.3125 mm, slices = 48, thickness = 3.3125 mm. 140 temporal volumes were acquired for each participant in a single scanning session. All data available from the ADNI database was acquired in accordance with the recommendations of local IRBs with written informed consent from all subjects. All subjects gave written informed consent in accordance with the Declaration of Helsinki. The protocol was approved by local IRBs. More specific information can be obtained from the ADNI website (http://www.loni.ucla.edu/ADNI). The data was subjected to a standard resting-state preprocessing pipeline using SPM12 (Friston et al., 1995) and DPARSF toolboxes (Chao-Gan and Yu-Feng, 2010), including slice timing correction, realignment and motion correction, normalization to MNI space, and spatial smoothing with a Gaussian kernel of 4 × 4 × 4 mm^3^ full width at half maximum (FWHM). Six rotation and translation parameters were first tested individually. Except rotation in Y axis (*P* < 0.05), there were no significant differences between the groups (*P* > 0.05). Then, all the six head motion parameters were aggregated into a single metric (i.e., framewise displacement), and no significant differences in framewise were found between the groups (*P* > 0.05). Nuisance variables such as the mean white matter signal, mean cerebrospinal fluid signal, and six head motion parameters were regressed out of the BOLD time series. It should be noted that band-pass filtering was not performed during pre-processing since it will likely impact deconvolution. Mean time series were extracted from 200 functionally homogeneous ROIs identified via spectral clustering (Craddock et al., 2012).

### Connectivity analysis

SEC was obtained using Granger causality (GC) analysis. However, before GC analysis is performed, it is necessary to acknowledge the impact of hemodynamic response function (HRF) on connectivity modeling, which is known to vary across different regions within a participant, as well as vary across participants (Handwerker et al., 2004). Previous studies have shown that results obtained by using GC analysis on HRF-corrupted fMRI data can be confounded by the variability of the HRF (David et al., 2008; Deshpande et al., 2010b). Hence, a blind deconvolution technique, proposed by Wu et al. (2013), was employed to minimize the non-neural variability of the HRF and estimate the latent neuronal time series from the observed fMRI data. In brief, the resting-state data was modeled as spontaneous event-related data (Tagliazucchi et al., 2012), and the HRF of each voxel was estimated by Wiener deconvolution (Glover, 1999). The estimated neural time series were then used in further GC analysis.

The underlying concept of GC is that a directed causal influence from time series *X* to time series *Y* can be inferred if the past values of time series *X* improves the prediction of the present and future values of time series *Y* (Granger, 1969). Let *q* time series *X*(*t*) = [*x*_1_(*t*), *x*_2_(*t*),…,*x*_*q*_(*t*)] be the latent neural time series obtained after HRF deconvolution of selected ROI fMRI time series, with *q* being 200 ROIs in this study. Then the multivariate autoregressive (MVAR) model with order *p* is given by

(1) X(t)=A(1)X(t−1)+A(2)X(t−2)​+⋯+A(p)X(t−p)​+E(t)

Where *A*(1)…*A*(*p*) are the model parameters, and *E*(*t*) is the vector of the residual error.

To remove the zero-lag correlation effect (i.e., ignore co-activations), the time series were input into a modified multivariate autoregressive model which included the zero-lag term used by Deshpande et al. (2009) shown as follows:

(2) $$X(t)=A^{\prime }(0)X(t)+A^{\prime }(1)X(t-1)+\cdots +A^{\prime }(p)X(t-p)+E(t)$$

The diagonal elements of *A*′(0) were set to zero, to model only the instantaneous cross-correlation rather than zero-lag auto-correlation. The off-diagonal elements of *A*′(0) corresponded to the zero-lag cross-correlation (Deshpande et al., 2009). It is to be noted that the coefficients in Equation (1) *A*(1),…*A*(p) would not be the same as *A*′(1)…*A*′(*p*) as in Equation (2), because the modified zero-lag term affects other coefficients since it removes the zero-lag cross correlation effects from them. Accordingly, the correlation-purged granger causality (CPGC) from time series *i* to time series *j* was obtained using the following equation

(3) $$CPGC_{ij}=\sum_{n=1}^{p}{\left(a\prime_{ij}\right)}^{2}(n)$$

Where $a_{ij}^{\prime }$ are the elements of *A*'. It is well-known that the coupling among brain areas is time-varying and context-sensitive. Indeed, the most interesting parameters of dynamic causal models are the fluctuations in effective connectivity (induced by experimental manipulations or time). In recent years, the functional connectivity (resting state) community has dubbed these fluctuations in coupling as “dynamic functional connectivity.” In our work, we characterized DEC using a temporally adaptive modified MVAR model:

(4) $$\begin{array}{l} X(t)=A^{\prime }(0,t)X(t)+A^{\prime }(1,t)X(t-1)+\cdots +A^{\prime }(p,t)X(t-p)\\ \;+E(t) \end{array}$$

In this model, the coefficients *A*′(*p*) were allowed to vary over time, thus “dynamically” estimating EC.

The parameters *A*′(*n*,*t*), *n* = 0,…,*p* were estimated in a Kalman filter framework using variable parameter regression (Arnold et al., 1998; Büchel and Friston, 1998). The Kalman filtering is a recursive process, where new information is added when it arrives. Thus, estimates taken from early steps are less reliable compared to later ones. A forgetting factor (FF) is introduced to circumvent this problem by taking recent past Kalman filter estimates into account during current estimation in order to control smoothness and enhance stability. The forgetting factor was determined by minimizing the variance of estimated error energy (Havlicek et al., 2010) and was found to be equal to one in our study. In brief, Kalman filtering treats the underlying MVAR coefficients as slowly fluctuating states. This enables the estimation of time varying directed connectivity that was used for subsequent modeling at the between-subject level. The DGC is estimated as:

(5) $$DGC_{ij}(t)=\sum_{n=1}^{p}{\left(a\prime_{ij}\left(n,t\right)\right)}^{2}$$

Where *DGC*_*ij*_ *(t)* is the dynamic Granger causality value from time series *i* to time series *j* at time point *t*. Given that the neural delays of interest are of the order of a TR or less (Deshpande et al., 2013), and that previous literature supports using a first order model to capture most relevant causal information (Deshpande and Hu, 2012), we employed a first order model for estimating both SEC and DEC in this work.

### Identification of disease foci

Connectivity studies often report aberrations in functional connections between brain regions. While this is useful, it does not provide a comprehensive characterization of the underlying connectomics. First, it is likely that several aberrations in connectivity are the after-effects arising from disruptions in certain focal brain regions. Second, our knowledge about brain functioning is centered on functions of regions rather than connections. Therefore, it is advantageous to identify certain focal regions of disruption using connectivity data. Thus in this study, we sought to identify diseased foci in AD. A recent study introduced a novel technique for the identification of disease foci (Venkataraman et al., 2013) based on non-directed FC differences between populations. Here we generalize this technique to the identification of diseased foci from effective connectivity as well as dynamic connectivity data.

**Figure 1:**
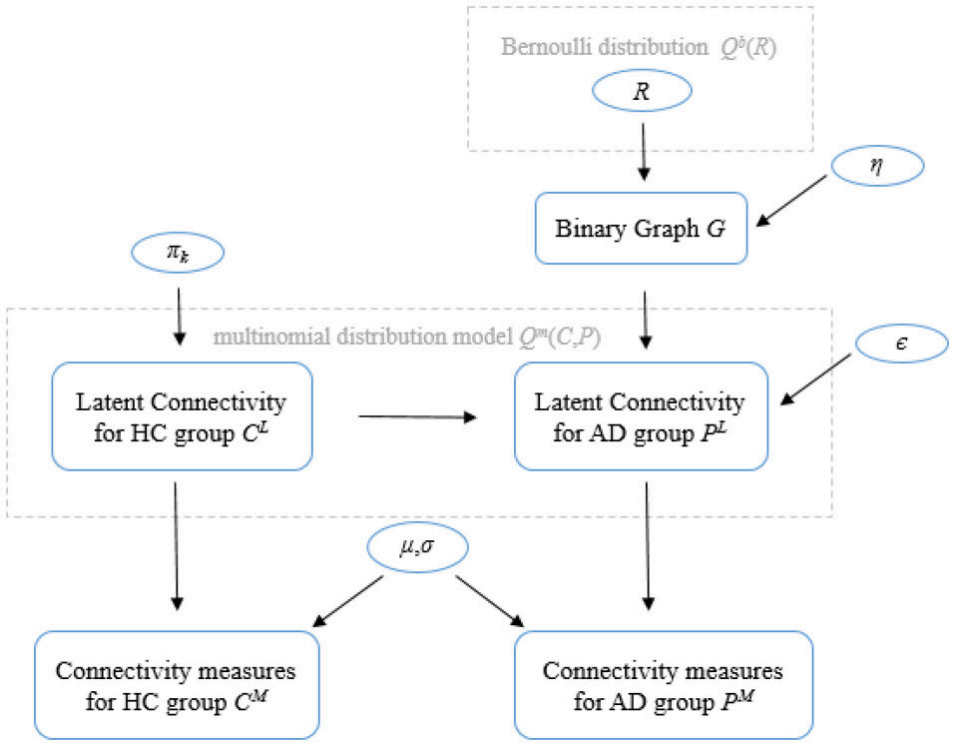
General model of the Foci identification technique. Parameters in circles indicate random variables. Please refer to the text for a description of the variables.

The model proposed by Venkataraman et al. (2012) considers the connectivity measure ($C_{ij}^{M}$ for HC group and $P_{ij}^{M}$ for the AD group) as a noisy observation of the latent connectivity ($C_{ij}^{L}$ for HC group and $P_{ij}^{L}$ for the AD group). The model is illustrated in Figure 1 and consists of several parts.

The first part defines a binary indicator vector that selects disrupted regions, and a binary graph characterizes corresponding abnormal connectivity. Let *N* be the total number of regions in the brain being considered. The model assumes a the random variable *R* = [*R*_1_,…,*R*_*N*_] is a binary vector (i.e., brain regions are either healthy with *R*_*i*_ = 0 or disrupted with *R*_*i*_ = 1, where *i* = 1 .. *N*) indicates the state of each region in the brain. Elements of *R* follow an independent, identically distributed (i.i.d.) Bernoulli distribution model *Q*^*b*^(*R*) where *Q*(^.^) denotes the posterior distribution and superscript *b* indicates a Bernoulli distribution. Then, an underlying binary graph *G* which characterizes the network of abnormal connectivity can be defined as follows: a connection between two healthy regions is always healthy with probability equal to 1, a connection between two disrupted regions is always abnormal with probability equal to 1, and a connection between a healthy region and a disrupted region is abnormal with probability η. The second part specifies the latent connectivity for controls (*C*^*L*^) as a tri-state variable from a multinomial distribution with parameter π_*k*_ (*k* denotes three different states), positive connectivity with probability π_1_, little or no functional connection (0) with probability π_0_, and negative connectivity with probability π_−__1_. Given the binary graph *G* and latent connectivity for controls *C*^*L*^, the tri-state latent connectivity of the AD population can be defined. Specifically, the latent connectivity from the control group $C_{ij}^{L}$ equals to $P_{ij}^{L}$ with probably ϵ if the binary graph connection between regions *i* and *j* is abnormal, $C_{ij}^{L}$ equals to $P_{ij}^{L}$ with probably 1 − ϵ if the connection between regions *i* and *j* is healthy. The third part characterizes the observed connectivity measures $C_{ij}^{M}$ and $P_{ij}^{M}$ as Gaussian random variables whose mean and variance (μ and σ) depend on the value of $C_{ij}^{L}$ and $P_{ij}^{L}$_._ Then, the joint likelihood of all configurations of latent connections between regions can be modeled as an 9-state multinomial distribution model *Q*^*m*^(*C, P*) (superscript *m* denotes that *Q*(^.^) is a multinomial distribution).

The model in Venkataraman et al. (2013) was applied in the case of functional connectivity, i.e., the Pearson's correlation coefficient between regions. However, EC is not a bounded measure, a small number of outliers is to be expected. In our EC data, we found a small portion of connectivity values which were >1 or < −1 (0.3%), wherein these outliers indicate stronger causal information flow between regions. To maintain the importance of those stronger effective connections and minimize its negative impact on model evaluation, inverse Fisher transformation was used to render the EC values as a bounded measure within [−1 1]. For the variance of dynamic EC, the latent tri-states of variance of connectivity v*F*_*ij*_ can be considered as follows: little variability or stationary connection, modest variability and strong variability. It is to be noted that static FC is direction-less, hence only the upper or lower triangle of the symmetric connectivity matrices were needed to fit the model in Venkataraman et al. However, in our case, both SEC and vDEC are directed with asymmetric connectivity matrices, and hence the whole matrices were used in the model. Taken together, these modifications permitted the model to be applied to both static and dynamic EC.

**Figure 2:**
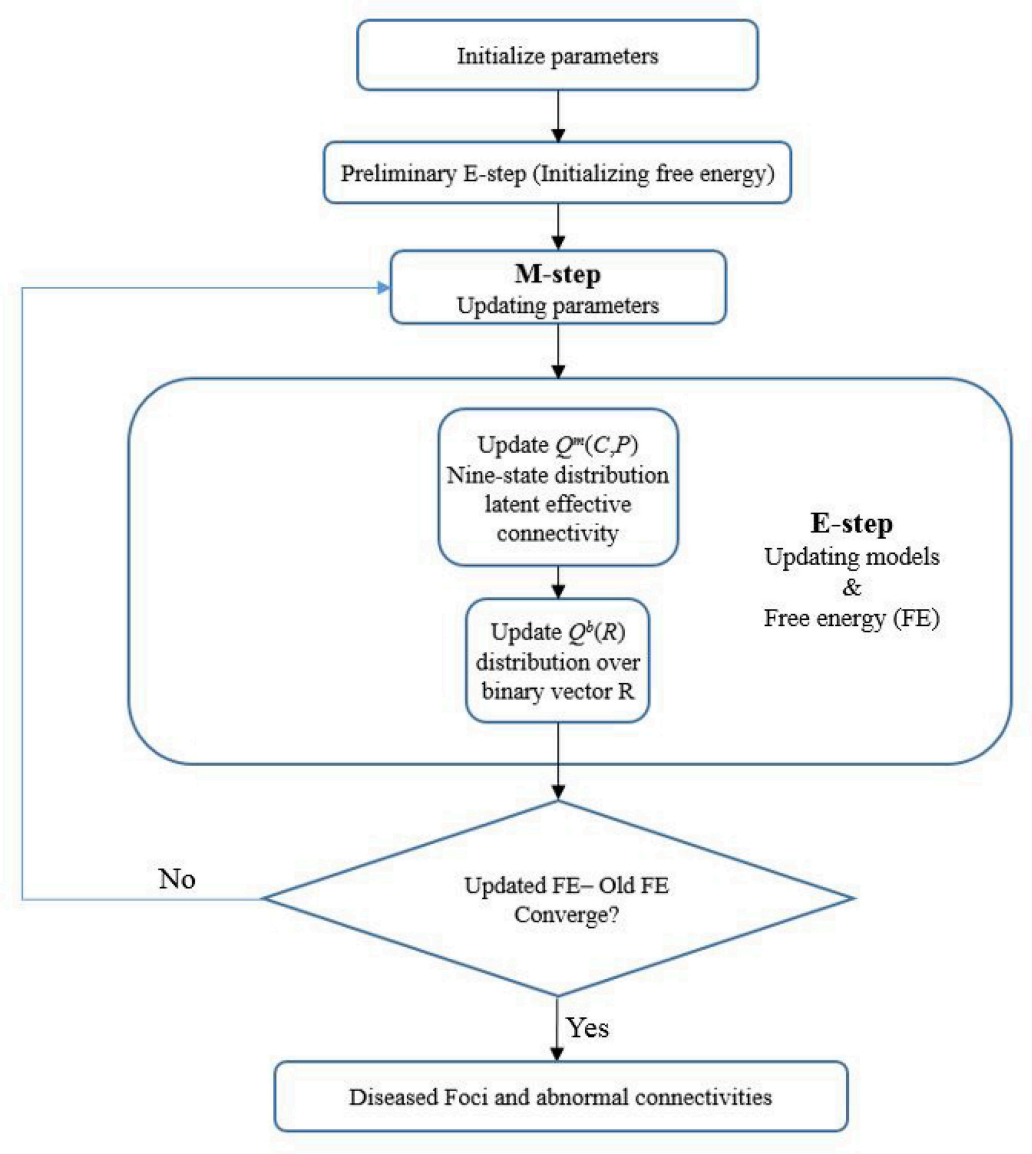
A flow chart of the Foci identification technique. The foci-identification technique posits that the latent connectivities can be stochastically generated from a distribution mode, and that the observed connectivity data are a noisy measurement of the latent unmeasured connectivity. Latent variables of the model were randomly initialized, and the variational EM algorithm was used to obtain the posterior distribution Q (both the nine-state distribution of latent functional connectivity and distribution over binary vector R) and model parameters to minimize the variational free energy. Then the disrupted foci and corresponding dysfunctional connections can be identified.

After initiating the prior parameters (such as the Bernoulli prior for binary state vector *R*, prior for latent connectivity for controls π_*k*_, etc.) for the model, a variational expectation maximization (EM) algorithm (Dempster et al., 1977) was adopted for estimating the latent connectivity and model parameters from the observed connectivity measures (*C*^*M*^ and *P*^*M*^). Technically, we inverted the (between subject) model of disconnection using variational Bayes. This scheme is formally similar to an EM algorithm that uses a variational update for all the factors of an approximate posterior. These included an approximate posterior distribution over model parameters (π_*k*_, η, ϵ, μ, and σ), latent connectivity for both groups of subjects [*Q*^*m*^(*C, P*)] and regional pathology [*Q*^*b*^(*R*)]. In brief, this variational scheme optimizes the sufficient statistics of each marginal distribution or density with respect to variational free energy (FE), under the expected values of the remaining factors. The variational EM alternates between updating the latent posterior distribution and estimating the nonrandom model parameters. Convergence was based on the relative change in free energy of the model of <10^−4^ between consecutive iterations. Disrupted focal regions and latent abnormal connectivity would then be identified from the posterior probabilities for each region and each connection. Figure 2 illustrates the flow chart of the algorithm.

The significance of the resulting foci was estimated using nonparametric permutation tests. Specifically, the group label of each participant was randomly permuted for 1,000 times. For each permutation, we fit the data to the model and obtained the posterior probability of disrupted foci for each region. This provided an empirical null distribution from which the *p*-value of the significance was obtained. The method also identified the affected connections associated with the disrupted foci. Among such connections, we retained those that were also in accordance with our hypothesis (paths exhibit lower SEC, as well as lower vDEC of effective connectivity in AD compared to healthy controls with a threshold of *p* < 0.05).

## Results

**Figure 3:**
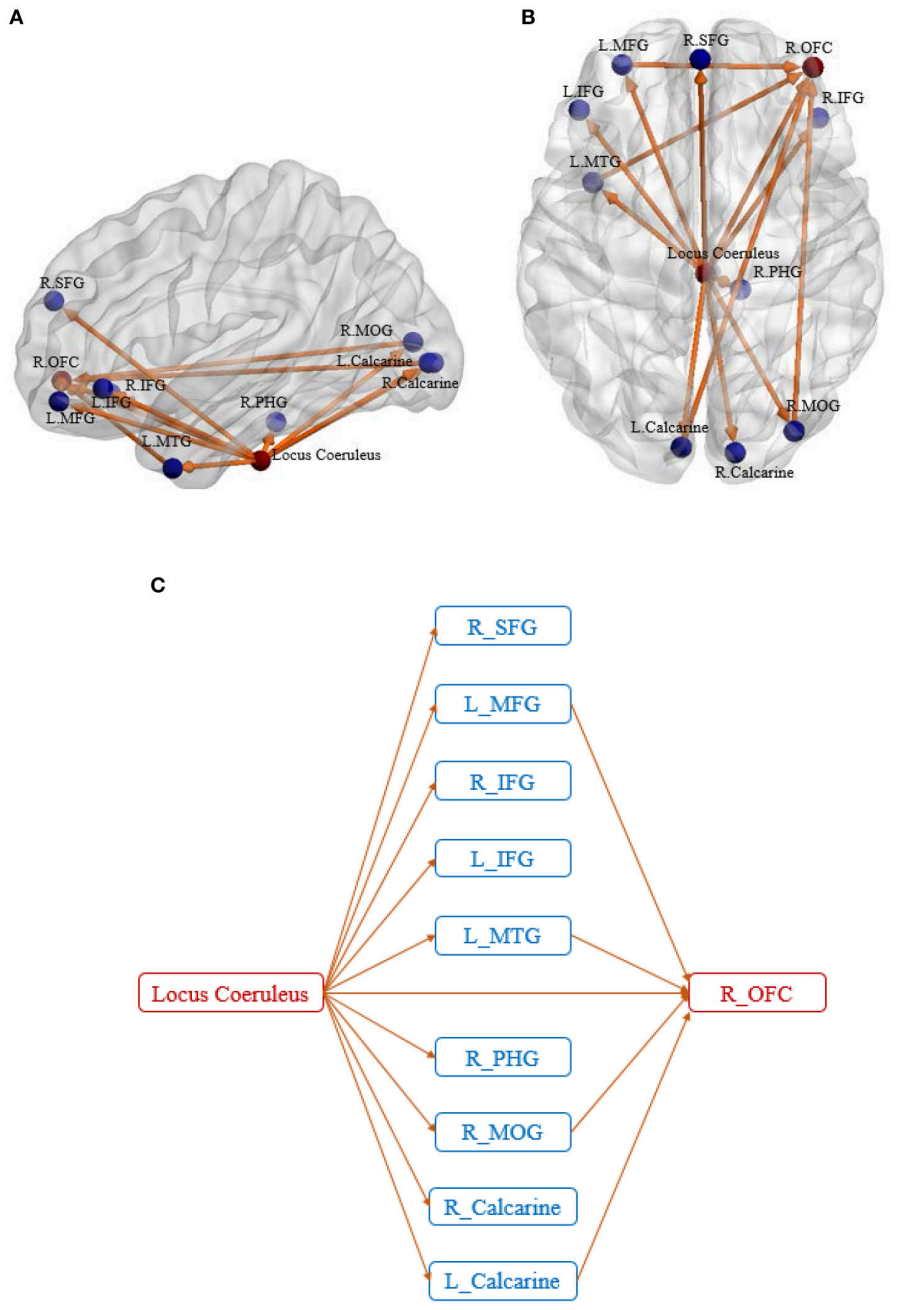
Sagittal view **(A)** and axial view **(B)** of the disease foci and corresponding disrupted connections. Regions in red are the identified affected foci, located in Locus Coeruleus and Right orbitofrontal cortex. Regions in blue are the non-foci regions that were connected from/to the disease foci. A schematic of the identified network is also shown for better visualization of the network architecture **(C)**. The expansions for the abbreviations are as follows: SFG, superior frontal gyrus; MFG, middle frontal gyrus; IFG, inferior frontal gyrus; MTG, middle temporal gyrus; PHG, parahippocampal gyrus; MOG, middle occipital gyrus; OFC, orbitofrontal cortex.

We identified two disrupted foci which were common to both SEC and vDEC networks: (1) Locus Coeruleus (LC) in the Brainstem (*p* = 0.003 for SEC and 0.006 for vDEC), (2) Right orbitofrontal cortex or R OFC (*p* = 0.007 for SEC and 0.002 for vDEC). Disrupted connectivity paths associated with these foci exhibited higher strength and larger temporal variability in HC as compared to AD (in accordance with our hypothesis). Furthermore, they exhibited a unique pattern of disrupted connectivity—those associated with the LC in the brain stem emanated from it, while connectivity paths associated with R OFC converged onto it (Figure 3).

Five of the ten connectivity paths emanating from the LC resulted in connectivity paths terminating in the R OFC, with four of these five paths being indirect pathways via the L MFG, L MTG, R MOG, and L Calcarine, and one path being a direct connection from LC to R OFC. All connectivity paths exhibited lower SEC and lower vDEC in AD compared to HC.

**Figure 4:**
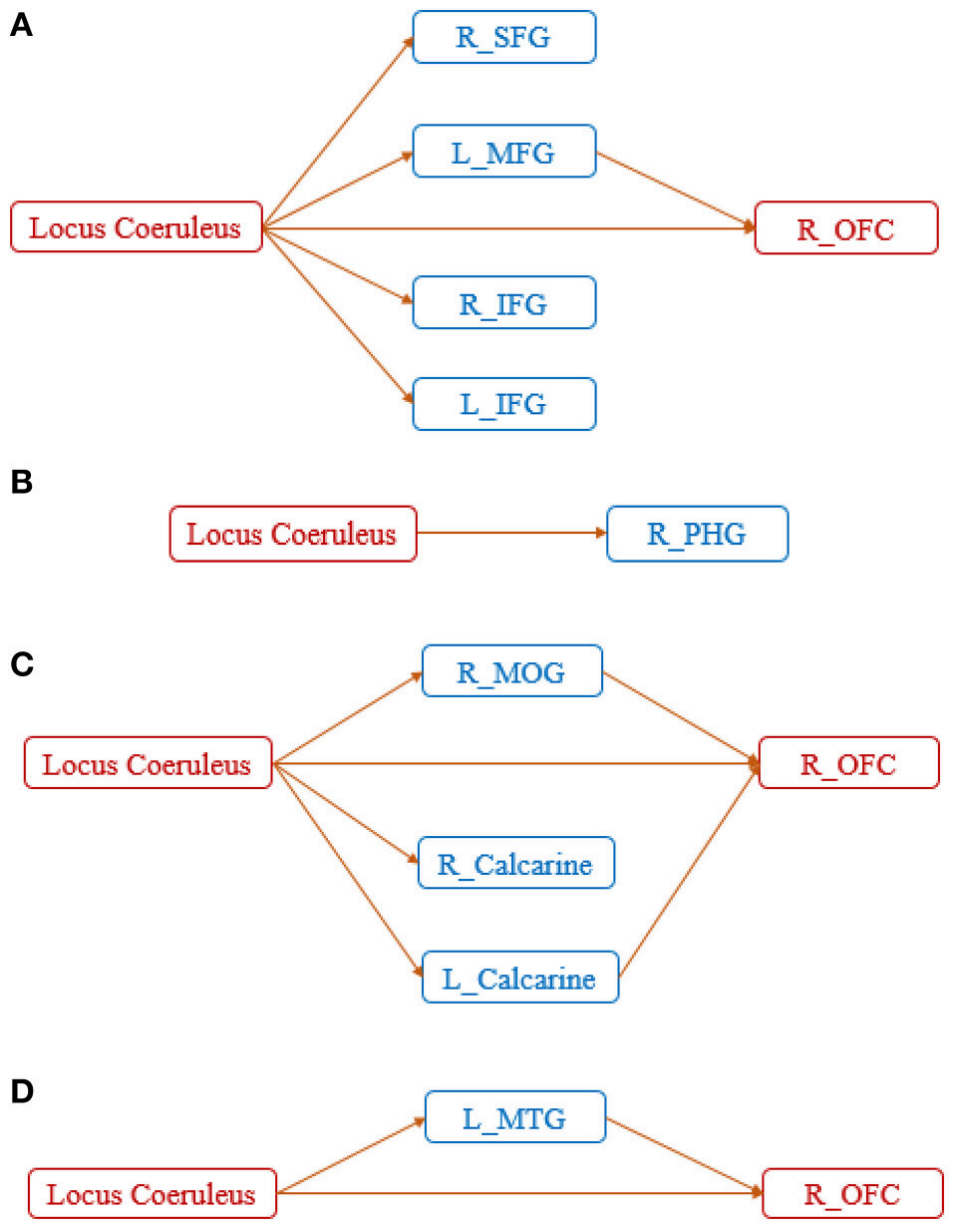
Disrupted networks associated with the diseased foci, showing the entire network partitioned into four unique subnetworks: **(A)** LC-PFC working memory system, **(B)** LC-PHG emotional memory system, **(C)** LC-visual sensory system, and **(D)** LC-MTG language system. SFG, superior frontal gyrus; MFG, middle frontal gyrus; IFG, inferior frontal gyrus; MTG, middle temporal gyrus; PHG, parahippocampal gyrus; MOG, middle occipital gyrus; OFC, orbitofrontal cortex.

Further clarity on the corresponding aberrant connectomic network was obtained by partitioning the network into four unique subnetworks: (Figure 4A) LC-PFC working memory system, (Figure 4B) LC-PHG emotional memory system, (Figure 4C) LC-visual cortex sensory system, and (Figure 4D) LC-MTG language system. Note that this partitioning is based on different functions performed by the locus coeruleus—norepinephrine system and is not based on any analytical strategy. Taken together, the disruption of these networks likely leads to working memory deficits, difficulties in processing emotional memories, and several other symptoms commonly observed in those with AD. The relevance of these subnetworks to AD pathology are discussed in detail in the next section.

## Discussion

In this study, we estimated static and dynamic measures of directed influences between 200 ROIs covering the entire brain in both AD and HC participants taken from the ADNI database. SEC and vDEC connectivity data were fed into a probabilistic model to identify regions with focal connectivity deficits in AD, with the hypothesis that connections associated with those regions would be weaker in strength and lower in temporal variability (i.e., rigid) in AD. We identified two such foci, brain stem and orbitofrontal cortex, which were affected significantly by the disease. The aberrant connections emanating from LC suggested a widespread dysregulation originating from the brainstem, part of which terminated into the other focus (orbitofrontal cortex).

Interestingly, all connectivity paths corresponded with the directed influence of the LC (in the brain stem) on mostly cortical (and few sub-cortical) regions. This corroborates with previous studies that have shown progressive damage (Kienzl et al., 1999) in the brain stem during early periods of AD. Further, LC in the brain stem is the largest repository of Norepinephrine (NE) in the human brain (Herregodts et al., 1991). Noradrenergic neurons in LC have projections to several parts of the brain including olfactory, limbic, prefrontal, and other cortical regions (Sara, 2009; Sara and Bouret, 2012). NE is known to suppress neuroinflammation (Weinshenker, 2008). This purported role has been hypothesized to be a protective factor against AD. In fact, Heneka et al. (2010) showed that NE stimulation of mouse microglia suppressed Aβ-induced cytokine and chemokine production and increased microglial migration and phagocytosis of Aβ. Induced degeneration of the brain stem increased the expression of inflammatory mediators in amyloid precursor protein (APP)-transgenic mice and resulted in elevated Aβ deposition. Kelly et al. (2017) suggesting that the decrease of NE in the brainstem facilitates the inflammatory reaction of microglial cells in AD and impairs microglial migration and phagocytosis, thereby contributing to reduced Aβ clearance. The Aβ is the critical initiating event in AD, starting with the aberrant clearance of Aβ-peptides followed by consecutive peptide aggregation and disruption of neural activity (Selkoe, 2002). Moreover, a post-mortem study has found significant volume decreases in the LC during AD progression, highlighting the importance of this region in AD (Theofilas et al., 2016). These findings indicate that the depletion of NE in LC is an etiological factor in the development of MCI and progression to AD. The studies discussed above provide some basis for the important role of brainstem in AD. Further, an animal study has found that boosting NE transmission can lead to increased functional connectivity (Guedj et al., 2016), suggesting that the reduction of NE could potentially result in lower connectivity between LC and cortical regions.

Several previous studies have suggested that OFC may be important for understanding the mechanisms for putative spreading of AD pathology in the brain (Van Hoesen et al., 2000; Sepulcre et al., 2013). Robust correlation has been found between Aβ deposition levels and volume in the orbitofrontal area (Ishibashi et al., 2014). In fact, the amyloid precursor protein (APP) gene contains the sequence for the Aβ peptide, which is concentrated in the senile plaques (SPs) (Cras et al., 1991). During AD progression, the SPs appear first in the orbitofrontal and temporal cortices and later extend to the whole cortex (Braak and Braak, 1999). Further, SPs and Aβ deposition has been associated with reduced connectivity at the synaptic level (Yeh et al., 2011), suggesting a potential mechanism that might link SPs and Aβ deposition with directed connectivity estimated from fMRI. While we discuss the role of temporal regions later in this section, the findings presented above highlight the importance of the role of OFC in AD.

Connectivity paths from LC to the prefrontal cortex (PFC) in general, and OFC in specific (note that OFC is a region in the PFC), can be considered as an aberrant LC-PFC working memory system (Figure 4A). Given that many studies have referred to the PFC in general without specifying sub-regions, and hence we are going to use the same nomenclature in the ensuing discussion. Previous studies have indicated that NE is instrumental in enhancing working memory through actions within the prefrontal cortex (PFC). PFC underlies the encoding of task-relevant information in working memory (Baddeley, 2003), and it has been shown that damage to the noradrenergic innervation of the PFC impairs performance in working memory (Brozoski et al., 1979). The stimulation of α_2_-adrenergic receptors in the PFC of nonhuman primates has been shown to improve performance in working memory tasks (Li et al., 1999) while α_1_-adrenergic receptors impaired the working memory (Arnsten and Jentsch, 1997). α_2_-adrenergic receptors have a higher affinity for NE compared to α_1_-adrenergic receptors, thus under normal conditions, NE facilitates working memory performance via actions at α_2_-adrenergic receptors in general and also in the PFC. However, dysfunction in noradrenergic pathways emanating from LC may result in low PFC NE levels, affecting working memory (O'Rourke et al., 1994).

The connectivity from LC to PHG can be considered as a LC-PHG emotional and spatial memory system (Figure 4B). The LC-NE system modulates emotional memories, and studies have suggested that emotional memories induce the activation of LC and subsequent NE release (Weiss et al., 1980). Corticotropin-releasing hormone (CRH) receptors are known play an important role in the coordination of autonomic and electrophysiological responses associated with emotional memories (Koob and Bloom, 1985; Dunn and Berridge, 1990). CRH-immunoreactive fibers were observed in the LC, suggesting that CRH may modulate LC neuronal activity (Merchenthaler et al., 1982; Cummings et al., 1983). In fact, many studies (Valentino et al., 1983; Finlay et al., 1997; Jedema et al., 2001) have shown that CRH administered locally into the LC increases LC discharge activity and NE release in its terminal fields. Moreover, an abundant expression of CRH was found in PHG (Wong et al., 1994). The first sign of emotional memories was also observed in PHG, and was found to then gradually spread to PFC and other cortical regions (Sotiropoulos et al., 2011). On the other hand, PHG is known to be involved in spatial memory (Bohbot et al., 1998). Noradrenergic neurons within LC have widely distributed, ascending projections to the limbic system including PHG (Szabadi, 2013). Thus, the LC-NE system may help trigger the involvement of the PHG in spatial memory. An animal study has indicated that the LC-NE system is necessary for the acquisition of spatial memories (Gertner and Thomas, 2006). These evidence suggest that the decrease of NE in LC could likely cause dysregulation of the emotional and spatial memory system in the LC-PHG network.

Connectivity paths from LC to the frontal cortex, mediated by sensory visual regions, can be considered as a LC-visual sensory system (Figure 4C). Previous works in animal models have shown that the LC-NE system can alter receptive field properties such as velocity tuning, direction selectivity, etc. (Waterhouse et al., 1990; McLean and Waterhouse, 1994). Malfunction of the LC-visual sensory network may contribute to deficits in visual assessment (Johnson et al., 2012).

Connectivity paths from LC to the OFC mediated by MTG can be considered as a LC-MTG language system (Figure 4D). A previous study has shown decreased regional cerebral blood flow (rCBF) after ingestion of an α_2_-adrenergic agonist drug in the MTG (Swartz et al., 2000). Given that the noradrenergic system in the brain originates from LC, this suggests that there might exist a noradrenergic pathway between LC and MTG which is impaired in AD. The malfunction of the LC-MTG language system may cause language impairments often observed in AD (Ferris and Farlow, 2013; Szatloczki et al., 2015).

It is evident that most of the disrupted connectivity paths emanating from the LC in the brain stem drive OFC either directly or via other systems. OFC is known to play a critical role in memory, emotions, reward, as well as decision-making mechanisms (Rolls, 2004; Rempel-Clower, 2007). Disrupted connectivity paths that converge into the OFC were observed in three of the subnetworks, and could potentially underlie behavioral deficits in these domains.

Taken together, we identified LC in the brainstem and OFC as the foci of network disruption in AD. The dysregulation of LC-NE neurotransmission likely contributes to behavioral deficits observed in AD. In corroboration, previous literature has pinpointed the same regions (Heneka et al., 2010; Ishibashi et al., 2014) to be affected in AD. Our identification of the LC in the brain stem as the disease focus in AD supports these previous observations and suggests that functional MRI studies of AD, which have been largely cortico-centric (Dennis and Thompson, 2014; Li et al., 2014), must in future investigate the role of this structure in AD.

Previous studies have also identified some other regions to be crucial to AD pathology (Brier et al., 2014; Dai et al., 2015; Mutlu et al., 2016). In fact, our foci-identification technique did identify some of the regions reported in these papers. Specifically, we also identified parahippocampal gyrus, middle frontal gyrus, and precuneus as foci only considering DEC networks. Further, middle temporal gyrus, lateral occipital cortex and cerebellum posterior lobe were identified as foci in SEC networks. However, these regions were not identified as foci in both DEC and SEC networks. Acknowledging that previous studies reported regions as having significantly different static connectivity between the groups, in this study we only reported the foci and the associated connectomic network that were found as having impairments in both static and dynamic EC.

Next, we report a few noteworthy limitations of this work. We have based our interpretation on the efferent projections of neurotransmitters arising out of LC. We employed this logic since functional imaging studies of the brain stem (and LC) in AD are limited, with the existing literature employing functional imaging in AD being cortico-centric. However, we have not directly measured norepinephrine in the brain, as it is difficult to do so using MRI. Therefore, our results form the basis for a hypothesis regarding dysfunction in the noradrenergic pathways in AD. Future studies must employ other modalities such as positron emission tomography for *in vivo* imaging of noradrenergic pathways (not just NE deficits) in AD. This could potentially open up possibilities for therapeutic interventions in AD. Further, the proposed methodology of combining static as well as DEC analysis with probabilistic modeling for identifying dysfunctional foci and associated dysfunctional networks could provide novel insights into the pathophysiology of other brain-based disorders.

## Author contributions

GD and PL designed the study; AV and DR contributed analysis tools; SZ performed data analysis; All authors interpreted the results and wrote the paper.

### Conflict of interest statement

The authors declare that the research was conducted in the absence of any commercial or financial relationships that could be construed as a potential conflict of interest.
